# A two-year follow-up study of the PCV2 status of a Danish pig herd that was initially assumed to be PCV2-free

**DOI:** 10.1186/2055-5660-1-5

**Published:** 2015-06-18

**Authors:** Charlotte S Kristensen, Charlotte K Hjulsager, Lars E Larsen

**Affiliations:** 1 Danish Agriculture and Food Council, Pig Research Centre, Vinkelvej 11, DK-8620 Kjellerup, Denmark; 2 National Veterinary Institute, Technical University of Denmark, National Veterinary Institute, Frederiksberg C, Denmark

**Keywords:** PCV2, Pigs, Longitudinal study, PCV2 vaccination

## Abstract

**Background:**

A longitudinal study was carried out to investigate whether a herd that had previously tested negative for porcine circovirus type 2 (PCV2) by polymerase chain reaction (PCR) was free of PCV2 or whether the negative profiles indicate that the level of PCV2 varies over time.

**Results:**

In eight Danish herds that had initially tested negative for PCV2 by quantitative polymerase chain reaction (PCR), additional sampling was performed. Only one of the herds was still PCV2-negative in the additional sampling and was included in the study.

The herd was a finishing herd, subclinically infected with PCV2, but vaccinated against PCV2 to improve performance. The herd was monitored by taking blood samples every seventh week over a period of two years and was not found to be continuously negative for PCV2 by PCR. The first time PCV2 was detected by PCR, in May 2010, PCV2 vaccination had been withdrawn from the herd, and at the same time the herd was infected with porcine reproductive and respiratory syndrome virus (PRRS). The PCV2-negative status, measured by PCR, was obtained in the first sampling after the PCV2 vaccination had been reintroduced. When PCV2 vaccination was withdrawn again in September 2011, the herd tested positive for PCV2 by PCR, and this time it continued to be PCV2-positive, even though PCV2 vaccination had been reintroduced.

**Conclusion:**

A Danish finishing herd that appeared to be PCV2-free from the start of a period of two years was not free of PCV2 during the entire period.

## Background

Porcine circovirus type 2 (PCV2) is required for the development of post-weaning multisystemic wasting syndrome (PMWS), a disease that causes significant financial losses to the pig industry globally [1]. Nowadays, most pigs become infected with PCV2, although only a few of them develop PMWS [1]. Several other disease syndromes, such as reproduction problems, diarrhoea and pneumonia, have also been linked to PCV2 infection, but this has been sustained only for reproduction problems [2, 3].

Under field conditions, the incubation period of PMWS is two to three weeks, and PCV2 can be detected in serum for up to 100 days after infection. Antibodies against PCV2 can be detected approximately two weeks after infection and persist for the lifetime of the slaughter pigs [4–7].

The predominant signs of PMWS were previously characterised by a high mortality rate among eight- to 12-week-old pigs. In order to diagnose PMWS, detailed histopathological and virological investigations are needed, and therefore dead pigs need to be submitted to the laboratory. Quantitative polymerase chain reaction (PCR) is widely used as a diagnostic alternative, since a PCV2 load above 7 log_10_ PCV2 copies/mL serum has been shown to be linked to the clinical and pathological appearance of PMWS [4–7].

During recent years, the nature of the disease has changed, and presently the signs are less severe and include moderately increased mortality, lower weight gain and increased prevalence of unspecified clinical signs in both weaners and finishers. Since the manifestations are seen also in finishers, all the diseases caused by PCV2 have been categorised as porcine circovirus diseases (PCVD) [1]. The clinical signs are very unspecific, and therefore PCVD remains a diagnostic challenge. In Denmark, many veterinarians use serum profiles to clarify whether a given herd problem is influenced by PCV2 circulation. The profile typically consists of ten samples from each of three groups (30, 70 and 100 kg) of growing pigs analysed by three PCR pools and 30 individual enzyme-linked immunosorbent assays (ELISA). With 30 samples, it should be possible to detect a prevalence of 10% with 95% certainty, independently of the number of pigs present in the herd.

In 2009, serum profiles from nearly 200 herds were submitted to the National Veterinary Institute, Technical University of Denmark (DTU) to be analysed for PCV2. A review of the results revealed that PCV2 was not detected by PCR in 28% of the serum profiles. The fact that PCV2 was not detected was somewhat surprising, since the serum profiles were taken from herds suspected of having a PCV2 problem among pigs with clinical signs of PCVD. Moreover, all herds were suspected of being infected with PCV2 [1].

The purpose of this study was to monitor herds that initially appeared to be PCV2-negative, measured by PCR, over time.

## Results

### Detection of PCV2 by PCR

The results of the PCR analysis in the two-year follow-up period are summarised in Table 1. In January and March 2010, the pigs tested negative for PCV2 by PCR. In May 2010, blood samples from pigs in the batches “pigs weighing 45 kg” and “pigs before slaughter” were positive for PCV2 by PCR. In 2011, all samples tested negative for PCV2 until May, when one pig tested PCV2- positive by PCR in the batch “pigs before slaughter”. In September 2011, one pig out of ten in the batch “pigs weighing 45 kg” tested PCV2-positive by PCR and, when that batch of pigs reached slaughter weight, ten out of ten pigs were PCV2-positive by PCR. In the remaining samplings (December 2011 and January 2012), the pigs in the batch “pigs before slaughter” remained positive for PCV2 by PCR (Table 1).

**Table 1:** **Average amount of PCV2 virus in pooled blood samples from pigs during a two-year period (log10 PCV2 copies/mL)**

	10 pigs weighing 45 kg		10 pigs before slaughter	
	PCV2 vaccinated	PCV2 virus load (no. positive/no. tested)	PCV2 vaccinated	PCV2 virus load (no. positive/no. tested)
2010				
January	Yes	neg^*^	Yes	neg
March	No	neg	Yes	neg
May	Yes	10^4**^	No	10^7**^
June	Yes	neg	Yes	neg
August	Yes	neg	Yes	neg
October	Yes	neg	Yes	neg
November	Yes	neg	Yes	neg
2011				
Janaury	Yes	neg	Yes	neg
February	Yes	neg	Yes	neg
April	Yes	neg	Yes	neg
May	Yes	neg	Yes	10^6^ (1/10)
July	Yes	neg	Yes	neg
September	No	10^5^ (1/10)	Yes	neg
November	Yes	neg	No	10^6^(10/10)
December	Yes	neg	Yes	10^5^ (3/10)
2012				
January	Yes	neg	Yes	10^5^ (2/10)

*Negative: the amount of PCV2 was below the detection limit (<3 log10 copies/mL).

**Samples from 2010 were not investigated individually.

All the additional samples taken in October 2010 (oral fluid samples, pen floor samples, cheek swabs, additional blood samples and dust samples) tested negative for PCV2 by PCR.

### Detection of PCV2 antibodies

The level of PCV2 antibodies in the herd was very low during most of the period (titre 0-50) for pigs immediately before slaughter. In November 2011, however, the level of antibodies was high (1250-31250) (data not shown).

## Discussion and conclusions

It was the intention to include more than one herd in the study, but it was difficult to find herds that had tested negative for PCV2 by PCR on more than one occasion, even though the serum profiles submitted to DTU indicated that 28% were negative. This could be due to lack of diagnostic submissions, low infectious pressure or lack of guidelines for veterinarians concerning which age groups were the most optimal to sample. It could also be due to the fact that there was particular emphasis on PCV2 causing reproductive problems, resulting in a large number of samples from sows.

The herd included in the study tested PCV2-negative by PCR in the beginning of the project, although subsequent samplings revealed that the PCV2 level measured by PCR fluctuated over time. Thus, in May 2010, blood samples from pigs weighing 45 kg and from pigs immediately before slaughter were positive for PCV2 by PCR. The pigs in the batch "pigs before slaughter" were not vaccinated against PCV2 at that time. At the same time, the pigs in the batch "pigs weighing 45 kg" had been born at a time when there was an acute outbreak of PRRS in the sow herd, and therefore the herd was infected with PRRS. It is not clear whether the increased level of PCV2 in the herd at this sampling was due to the lack of PCV2 vaccination or whether it was triggered by PRRS, which has been shown to be a cofactor of PMWS [8–10]. However, once the PCV2 vaccination was resumed, tests from the herd turned out negative for PCV2 measured by PCR, even though PRRS infection transmission (high titre against PRRS in an indirect immunoperoxidase monolayer assay, results not shown) remained active until December 2010. In May 2011, one pig tested PCV2-positive by PCR in the batch “pigs before slaughter”, even though all of the pigs had been vaccinated. In September 2011, the batch “pigs weighing 45 kg” was not vaccinated against PCV2 and immediately tested PCV2-positive by PCR. In the following month, the batch “pigs before slaughter” seemed to be continuously PCV2-positive by PCR, even though PCV2 vaccination had been resumed.

With 30 samples, it should be possible to detect a prevalence of 10% with 95% certainty, independently of the number of pigs present in the herd. However, to increase the certainty and detect an even lower prevalence of PCV2, additional samples were taken in October 2010. All of the samples (a total of 45 blood samples, 45 cheek swabs, 45 dust swabs, nine oral fluid samples and nine faeces floor samples) were negative for PCV2 by PCR, indicating that it was not due to inadequate diagnostic evaluation that the herd tested negative for PCV2 by PCR.

The level of PCV2 antibodies in the herd remained very low during most of the period in pigs immediately before slaughter, which probably reflects antibody responses to the vaccinations. In contrast, the high antibody level in November 2011 was probably a response to an active infection since the pigs also tested virus-positive by PCR.

The sows were continuously vaccinated against PCV2 with Ingelvac®CircoFLEX before each farrowing. Although this is an off-label use of the vaccine, it is commonly done in Denmark. It could be argued that the vaccination of the sows might interfere with the introduction of active immunisation in pigs at weaning. However, it has previously been shown that vaccination of piglets with the same vaccine as that used on their mothers’ sows does not appear to affect vaccine efficacy in piglets [11].

The conclusion drawn from monitoring a Danish finishing herd that appeared to be PCV2-free from the start over a period of two years was that the herd was not free of PCV2 during the whole period. For this reason, one should be careful when assessing the impact of PCV2 on the basis of a single serum profile in a herd. The results from this herd indicated that PCV2 vaccination may reduce the PCV2 infection level even during an acute outbreak of PRRS, but the results also showed that PCV2 vaccination does not eradicate the virus from the herd. Further studies are needed to draw general conclusions on this matter.

## Methods

### Selection and screening of herds

Serum samples from a total of 196 herds were submitted to DTU for PCV2 analysis by PCR in 2009. The collection of samples from the 196 different herds was distributed over the year, with between one and 50 samples submitted from each herd. In 54 (28%) of these herds, PCV2 could not be detected in serum by PCR [11]. Eight herds that had initially submitted at least ten blood samples from finishers that had not submitted blood samples in relation to a project and herds whose owner was interested in participating in the project were selected among the 54 herds. Ten blood samples from finishers weighing 30 kg, 45 kg and 90 kg, respectively, were taken in these eight selected finishing herds to confirm their negative status. A total of 30 samples were taken from each herd, resulting in 240 samples. The samples were tested by PCR for PCV2 in pools of ten (24 pools in total).

Of the eight herds, seven tested PCV2-positive by PCR in the follow-up PCV2 serum profile consisting of 30 samples/herd. Therefore, only one herd was included in the study in January 2010.

### Herd description

In the herd, one thousand 7 kg pigs were purchased from the same supplier every seventh week. In the herd supplying these pigs, all sows were vaccinated against PCV2 (Ingelvac®CircoFLEX, 1 mL) three weeks prior to each farrowing. Additionally, all pigs were vaccinated against PCV2 (Ingelvac ®CircoFLEX, 2 mL) at weaning at four weeks of age at start of the study. The signs of PCVD were subclinical, and the purpose of the vaccination was to increase productivity. The herd was located at two sites in the southern part of Jutland. From 7 to 30 kg, all 1000 pigs were located at site I in a large pen with straw bedding. This site was managed all-in/all-out. From 30 to 100 kg, the pigs were moved to site II, which consisted of nine well separated sections, also managed all-in/all-out, housing 2000 pigs, and a tenth isolated section, which was used as a buffer section. Site II contained two batches of pigs with an age difference of seven weeks. Because of the substantial age difference, older pigs were never mixed with younger pigs. Two batches of finishers (placed at site II in May 2010 and in September 2011) were not vaccinated against PCV2 in order to evaluate the benefit of vaccination. In May 2010, the herd was infected from the sow herd with type 2 porcine reproductive and respiratory syndrome (PRRS). By December 2010, the herd was once again receiving pigs from the supplier that were PRRS-negative, based on ELISA tests.

### Sample collection

The herd included in the study was monitored every seventh week during a 2-year period. Each time, blood samples were taken from ten pigs weighing 45 kg (mid-finishing) and ten pigs immediately before slaughter. The blood samples were taken randomly from the appropriate age group. Serum profiles were taken 16 times in total, resulting in 320 samples.

On one occasion, in October 2010, several additional samples were tested to ensure that the lack of PCV2-positive results was not due to an insufficient number of samples. The following test samples were taken from a selected pen in each of the nine sections:

- Oral fluid sample
- pen floor sample (by hand, pool of five portions of faeces)
- five blood samples from pigs
- five cheek swab samples from pigs

Five dust samples were also taken from the inventory in each section using cotton swabs.

### Laboratory analysis

The amount of PCV2 was determined using real-time quantitative PCR on samples that had been pooled per age group (ten samples/age group) [12]. The effect of pooling has been validated on field samples and shown to have limited effect on the sensitivity and specificity of the test (unpublished results), as has been shown by others [13]. The detection limit of the PCR assay used was 3 log_10_ PCV2 copies/mL serum [12]. If a pool tested positive, the samples were tested individually by PCR (this only applies to samples taken in 2011 and 2012). The samples taken from pigs immediately before slaughter were also analysed individually for antibodies against PCV2 by an in-house ELISA [8].
